# Comparison of three lymph node staging systems in evaluating the prognosis of patients with pT3 esophageal squamous cell carcinoma

**DOI:** 10.1038/s41598-020-74327-y

**Published:** 2020-10-13

**Authors:** Di-tian Liu, Lin-shuo Wang, Yu-ping Chen, Shao-bin Chen

**Affiliations:** Department of Thoracic Surgery, Cancer Hospital of Shantou University Medical College, 7 Raoping Road, Shantou, 515000 Guangdong China

**Keywords:** Oesophageal cancer, Surgical oncology

## Abstract

To explore the prognostic value of three lymph node staging systems, including number of positive lymph nodes (pN), lymph node ratio (LNR), and log odds of positive lymph nodes (LODDS), in patients with pT3 stage esophageal squamous cell carcinoma (ESCC). Data from 1667 patients with pT3 stage ESCC who underwent surgical resection were reviewed. The log-rank test was used to assess the differences in overall survival (OS) between groups. Multivariate analysis was performed to identify independent prognostic factors. The receiver operating characteristic curve was used to assess the prognostic accuracy of the three staging methods. The median survival time for the entire group was 48.0 months, and the 1-, 3- and 5-year OS rates were 83.9%, 55.1% and 66.6%, respectively. All three lymph node staging systems were significantly correlated with OS in univariate and multivariate analyses. However, LNR and LODDS staging systems could more accurately predict survival than the pN staging system in patients with < 15 lymph nodes dissected, while LODDS have the best prognostic homogeneity. All three staging systems could be used for prognostic assessment in pT3 stage ESCC. But LODDS staging system might be superior to the others due to its prognostic homogeneity.

## Introduction

Esophageal carcinoma is a major cause of cancer-related death worldwide^1^. Esophagectomy with appropriate lymphadenectomy remains the major component of therapy for resectable cases. Lymph node (LN) metastasis is one of the most important predictors of survival for these patients after curative resection. Currently, the most widely used staging system for esophageal carcinoma is the American Joint Committee on Cancer (AJCC) and the International Union for Cancer Control (UICC) tumor node metastasis (TNM) system, which classifies the pathologic nodal (pN) stage based on the absolute number of involved nodes. However, most previous studies have found that the accuracy of this nodal stage is easily influenced by the total number of LNs examined^2–5^. When the number of examined LNs is small, stage migration may occur, leading to understaging of nodal status.

Recently, other staging methods have been proposed to improve the prognostic accuracy of the nodal stage, such as the lymph node ratio (LNR) and the log odds of positive lymph nodes (LODDS). Previous studies have shown that such staging methods could be used for prognosis evaluation in many malignancies and might even be better than the number-based pN stage^3–11^. However, few studies have compared these nodal staging methods in esophageal carcinoma^12^. Moreover, no widely accepted criteria have been established for LNR and LODDS staging. Our study was aimed to evaluate the prognostic value of three nodal staging systems (pN, LNR, and LODDS) in patients with pT3 stage esophageal squamous cell carcinoma (ESCC) after radical esophagectomy.

## Patients and methods

### Patients

This study was approved by the Ethics Committee of Shantou University Medical College Cancer Hospital. All methods were performed in accordance with the approved guideline. All participants signed an informed consent form before they entered this study. From January 1995 to December 2013, 4298 patients with esophageal cancer received surgery in our hospital. Only the patients who met the following criteria were evaluated in this study: (1) histopathologic diagnosis of ESCC; (2) radical surgery; (3) pathologic stage T3NxM0; and (4) no prior adjuvant therapy before surgery.

The pN stage was classified into four subgroups based on the 7th edition UICC/AJCC TNM staging system^13^: pN0 (no positive LNs), pN1 (1–2 positive LNs), pN2 (3–6 positive LNs), and pN3 (≥ 7 positive LNs). The LNR was determined by the ratio between metastatic and examined LNs. The LNR was classified into four subgroups based on the following intervals according to our previous study^4^: LNR0 (LNR = 0), LNR1 (0% < LNR ≤ 10%), LNR2 (10% < LNR ≤ 20%), and LNR3 (LNR > 20%). LODDS was calculated by the log (positive LN +  0.5)/(negative LN + 0:5). We used the same cutoff points previously reported by Sun et al.^14^: LODDS1 (LODDS ≤ − 1.5), LODDS2 (− 1.5 < LODDS ≤ − 1), LODDS3 (− 1 < LODDS ≤ − 0.5), and LODDS4 (LODDS > − 0.5).

### Surgery

Esophagectomy with lymphadenectomy was conducted via a left thoracotomy for most patients before 2010, while a right thoracotomy was routinely performed after 2010; thoracoscopic esophagectomy was also performed after 2011. A standard abdominal lymphadenectomy (left and right paracardial regions, along the lesser curve and the left gastric artery) and mediastinal lymphadenectomy (subcarinal, left and right bronchial, para-esophageal and thoracic duct, lower posterior mediastinum, pulmonary ligament) was performed in all patients. The left and right recurrent laryngeal nerve lymph nodes were also removed in patients underwent a right thoracotomy. Cervical lymphadenectomy was not systematically undertaken.

### Statistical analysis

Statistical analyses was analyzed using the SPSS 20.0 software (IBM, Armonk, New York, USA). Overall survival (OS) was determined by the Kaplan–Meier method. Survival differences was calculated by log-rank test. Multivariate analyses were conducted to investigate independent prognostic factors. Spearman’s correlation analysis was used to assess the relationship between different lymph node stages and the number of dissected lymph nodes. The receiver operating characteristic curve (ROC) was performed to assess the prognostic accuracy of the three staging methods. P < 0.05 was set as significance.

## Results

### Patient characteristics

The clinicopathological characteristics of 1667 patients with pT3 stage ESCC who met the inclusion criteria are shown in Table 1. This study group included 1226 men and 441 women with a median age of 56 years (range, 30 to 82 years). A total of 24,650 LNs were dissected with a median number of 14 (range, 4–69), and the mean number of metastatic nodes was 1.55 per case. Nine hundred fifty-six patients had fewer than 15 LNs retrieved, while 708 patients had 15 or more LNs retrieved. Four hundred and fifty-three patients received adjuvant radiotherapy, 301 patients received adjuvant chemotherapy, 49 patients received adjuvant radiochemotherapy, while the other 864 patients did not received adjuvant therapy.

**Table 1:** Univariate analysis for prognosis according to patient and tumor characteristics.

Variable	No. patients	MST (months)	1-year OS (%)	3-year OS (%)	5-year OS (%)	*P* value
**Gender**						0.001
Male	1226	43.0	85.6	53.6	45.0	
Female	441	69.0	87.8	60.8	52.8	
**Age (year)**						0.001
≤ 60	1096	55.0	86.2	57.5	49.2	
> 60	571	39.0	86.0	51.7	43.1	
**Tumor location**						0.366
Upper third	148	33.0	83.8	48.6	41.9	
Middle third	1236	50.0	87.1	56.6	47.6	
Lower third	283	49.0	83.4	54.4	47.7	
**Tumor length**						0.135
≤ 5 cm	965	51.0	86.8	56.3	48.2	
> 5 cm	702	45.0	85.2	53.8	45.6	
**Histologic grade**						< 0.001
Well	486	120.0	91.8	67.1	59.3	
Moderate	963	42.0	85.8	53.5	45.1	
Poor	218	23.0	75.2	38.5	28.9	
**Number of lymph nodes retrieved**						0.010
≤ 15	958	42.0	85.9	54.0	44.1	
> 15	708	65.0	86.4	57.5	51.1	
**pN**						< 0.001
pN0	827	124.0	92.7	70.1	61.5	
pN1	471	35.0	86.0	49.3	40.3	
pN2	288	18.0	72.2	30.9	23.3	
pN3	81	23.0	69.1	29.6	23.5	
**LNR**						< 0.001
LNR0	827	124.0	92.7	70.1	61.5	
LNR1	211	46.0	87.8	54.8	48.4	
LNR2	414	27.0	80.9	41.8	33.1	
LNR3	205	17.0	68.3	24.9	15.1	
**LODDS**						< 0.001
LODDS0	276	–	95.7	78.6	71.4	
LODDS1	617	89.0	90.8	64.8	56.4	
LODDS2	444	33.0	84.2	47.7	39.0	
LODDS3	330	19.0	72.1	29.1	20.3	

LODDS, log odds of positive lymph node; MST, median survival time; LNR, lymph node ratio; OS, overall survival.

### Relationship between different lymph node stages

**Figure 1:**
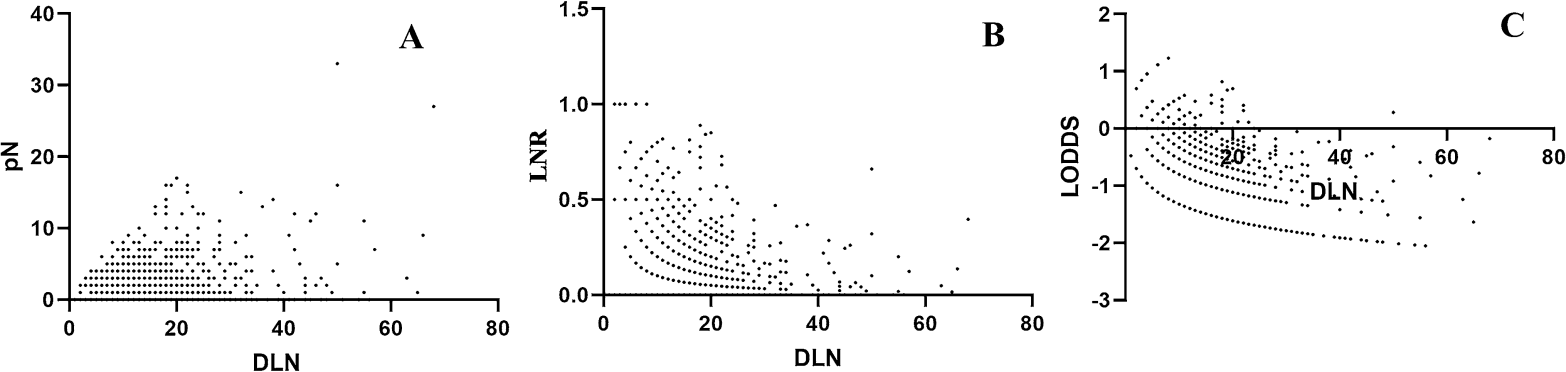
(**A**) The distribution of the number of lymph node metastasis (pN) and the number of dissected lymph nodes (DLN). (**B**) The distribution of lymph node ratio (LNR) and the number of DLN. (**C**) The distribution of log odds of positive lymph nodes (LODDS) and the number of DLN.

The relationships between three LN staging system and the number of dissected lymph nodes are shown in Fig. 1. Only the pN was correlated with the number of dissected lymph nodes (R = 0.136, P < 0.001), LNR (R = 0.018, P = 0.451) and LODDS (R = 0.025, P = 0.378) were not correlated with the number of dissected lymph nodes. Moreover, we compared the relationships between LODDS with the other two LN staging systems. LODDS was found to be more highly correlated with LNR than with pN (r = 0.970 versus r = 0.838), indicating that LODDS shared more similar properties with LNR than with pN.

### Survival and prognostic factors

Follow-up continued until December 2018. The mean follow-up period was 68.9 months (range, 1–272 months). Nine hundred ninety-five patients died, 620 patients survived, and 52 patients were lost to follow-up (3.1%).

**Figure 2:**
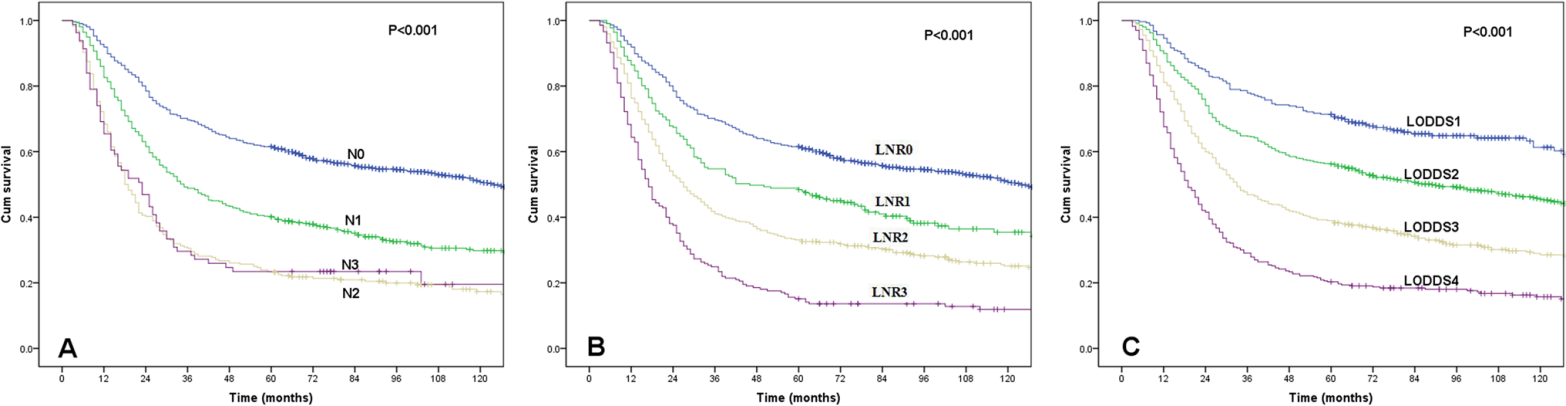
Kaplan–Meier curves for overall survival according to pN categories (**A**), LNR categories (**B**), and LODDS categories (**C**). All of the survival differences were significant (*P* < 0.001).

The median survival time (MST) for the entire group was 48.0 months (95% confidence interval (CI) 40.3–55.7 months), and the 1-, 3- and 5-year OS rates were 83.9%, 55.1% and 46.6%, respectively. The variables related to OS are shown in Table 1. The MSTs for the four pN categories (pN0-pN3) were 124.0, 35.0, 18.0, and 23.0 months, respectively, and the 5-year OS rates were 61.5%, 40.1%, 23.3%, and 23.5%, respectively (Fig. 2A, P < 0.001). However, the survival difference between the pN2 and pN3 categories was not significant (P = 0.335) in a separate subgroup analysis. The MSTs for the four LNR categories (LNR0–LNR3) were 124.0, 46.0, 27.0, and 17.0 months, respectively, and the 5-year OS rates were 61.5%, 48.4%, 33.1%, and 15.1%, respectively (Fig. 2B, P < 0.001). Subgroup analysis showed significant survival differences for all LNR categories. The MSTs for the four LODDS categories (LODDS1–LODDS4) were not reached, 89.0, 33.0, and 19.0 months, respectively, and the 5-year OS rates were 71.4%, 56.4%, 39.0%, and 20.3%, respectively (Fig. 2C, P < 0.001). Subgroup analysis also showed significant survival differences for all LODDS categories. Sex, age, histologic grade, and number of LNs dissected were also found to affect OS (P < 0.05).

The multivariate analysis incorporated variables that were significant in the univariate analysis, while pN (model 1), LNR (model 2), and LODDS (model 3) were studied separately, and combined three lymph node classifications in model 4 (Table 2). All three LN staging classifications were found to be independent prognostic factors in the separate analyses (P < 0.05), while only LODDS, but not pN and LNR, was independent prognostic factor in the combined analyses. Sex, age, and histologic grade were other prognostic factors in all four models, while the number of LNs dissected was only an independent prognostic factor in model 1 (P < 0.001). We furthermore compared the − 2log likelihood between these three lymph node staging system in multivariate regression analysis.The smaller the − 2log likelihood, the better the system. The − 2log likelihood for the pN, LNR, and LODDS were 14,274.590, 14,258.815, and 14,242.745, respectively, indicating that LODDS might be better than the other two lymph node stages.

**Table 2:** Multivariate analysis of prognostic factors in patients with pT3 stage ESCC.

	Hazard ratio	95% CI	P value
**Model 1**			
Gender	0.816	0.706–0.942	0.006
Age	1.294	1.141–1.168	< 0.001
Histologic grade	1.337	1.208–1.479	< 0.001
Number of lymph node dissection	1.275	1.123–1.448	< 0.001
pN	1.516	1.420–1.619	< 0.001
**Model 2**			
Gender	0.814	0.705–0.940	0.005
Age	1.301	1.147–1.476	< 0.001
Histologic grade	1.313	1.186–1.453	< 0.001
Number of lymph node dissection	1.106	0.975–1.254	0.116
LNR	1.436	1.358–1.517	< 0.001
**Model 3**			
Gender	0.819	0.709–0.946	0.006
Age	1.320	1.163–1.497	< 0.001
Histologic grade	1.341	1.213–1.483	< 0.001
Number of lymph node dissection	0.882	0.775–1.003	0.056
LODDS	1.573	1.473–1.681	< 0.001
**Model 4**			
Gender	0.818	0.708–0.945	0.006
Age	1.315	1.158–1.488	< 0.001
Histologic grade	1.346	1.216–1.486	< 0.001
Number of lymph node dissection	1.082	0.933–1.185	0.089
pN	1.036	0.943–1.138	0.443
LNR	0.953	0.864–1.051	0.326
LODDS	1.559	1.458–1.668	< 0.001

CI, confidence interval; LODDS, log odds of positive lymph node; LNR, lymph node ratio.

### Comparison of the prediction consistency between different LN classifications

The 5-year OS rates according to pN and LNR classifications stratified by LODDS were showed in Table 3. When stratified by the LODDS, significant survival differences could always be found among patients in each pN and LNR category, with the exception of the pN3, LNR2, and LNR3 categories. However, survival was more homologous when the LODDS classification was stratified by the pN or LNR.

**Table 3:** Comparison of 5-year overall survival rates with different pN and LNR classifications stratified by the LODDS.

	LODDS0	LODDS1	LODDS2	LODDS3	*P*^*a*^
**pN**					
pN0	71.4%	57.7%	46.2%	–	0.000
pN1	66.7%	49.5%	40.5%	23.0%	0.004
pN2	–	66.7%	30.9%	18.8%	0.044
pN3	–	–	40.0%	22.4%	0.628
*P*^*b*^	0.911	0.162	0.064	0.681	
**LNR**					
LNR0	71.4%	57.7%	46.2%	–	0.000
LNR1	66.7%	50.0%	37.4%	–	0.011
LNR2	–	–	34.7%	29.3%	0.122
LNR3	–	–	–	15.1%	–
*P*^*c*^	0.911	0.081	0.094	0.027	

LODDS, log odds of positive lymph node; LNR, lymph node ratio.

*P*^*a*^: Comparison of overall survival rates between different LODDS groups.

*P*^*b*^: Comparison of overall survival rates between different pN groups.

*P*^*c*^: Comparison of overall survival rates between different LNR groups.

**Figure 3:**
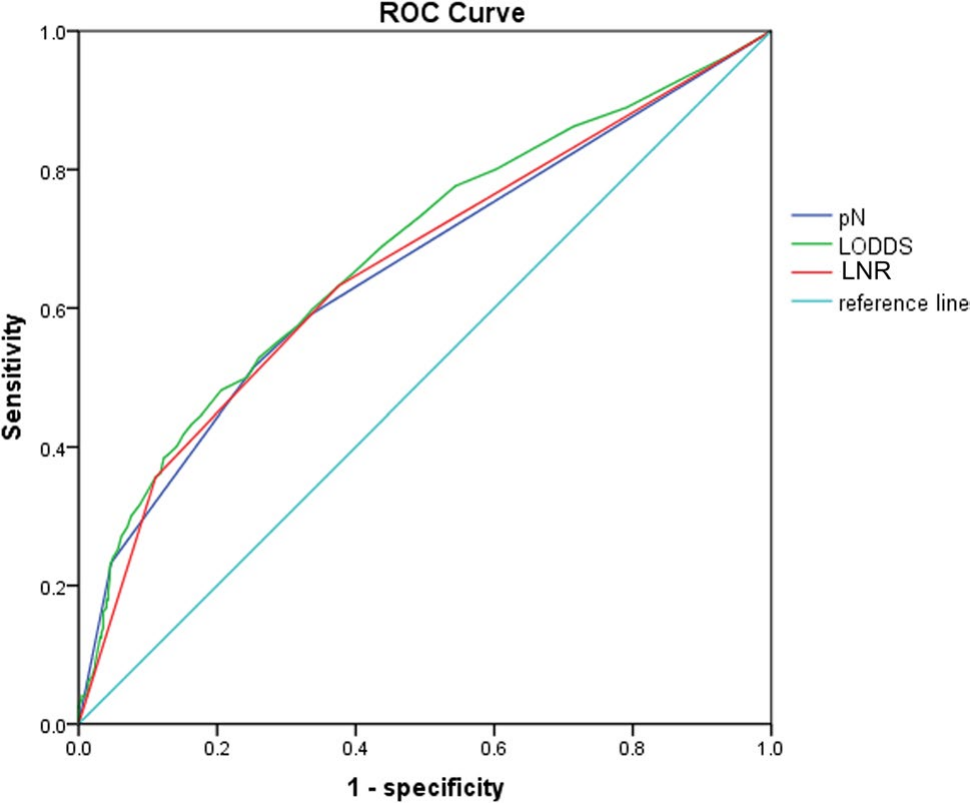
ROC curves of pN, LNR, and LODDS for predicting survival.

We further used the 5-year OS as the gold standard to draw ROC curve to assess the prognostic accuracy of these three LN staging systems. The corresponding area under the curve (AUC) for pN, LNR, and LODDS in the entire group was 0.671 (95% CI 0.645–0.697), 0.680 (95% CI 0.655–0.706), and 0.708 (95% CI 0.684–0.733), respectively (Fig. 3). The difference was not significant (P = 0.347).

**Figure 4:**
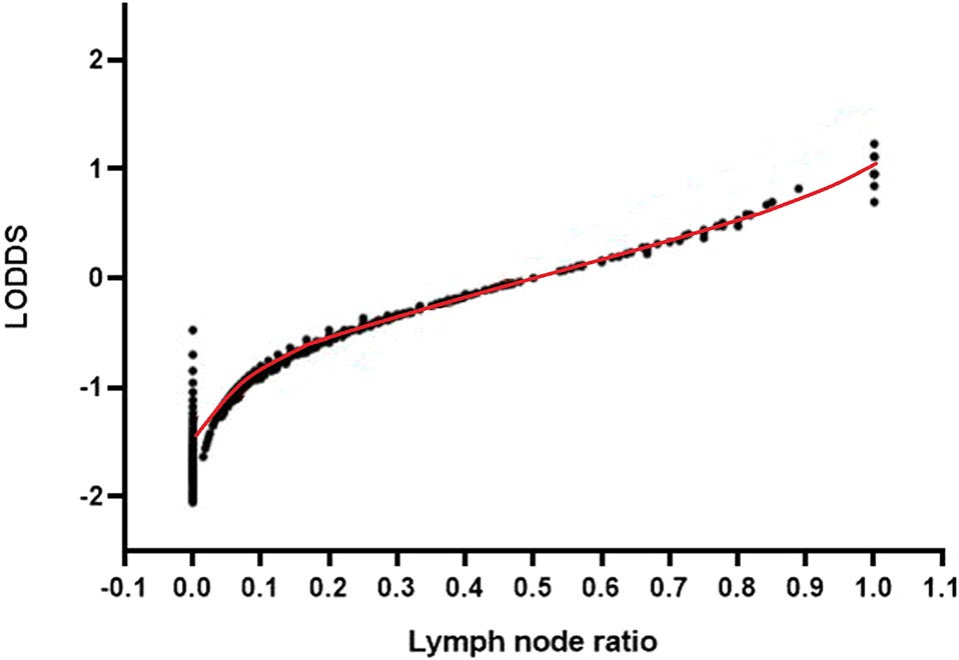
Scatter plots of the associations between LODDS and LNR.

We further plotted scatter plots of the relationship between LODDS and LNR to evaluate the superiority between these two staging systems (Fig. 4). There was consistent agreement between LNR and LODDS. However, when the value of LNR was equal to 0 or 1, the corresponding value of LODDS was quite heterogeneous, indicating that the LODDS has the potential to discriminate survival differences in patients without LN metastasis or with LNR = 1.

### Comparison of the prognostic accuracy of three LN stage in patients with different lymphadenectomies

When the patients in the pN0, pN1, pN2, and pN3 categories were subdivided by the extent of lymphadenectomy, those with ≥ 15 LNs examined had significantly better OS than those with < 15 LNs examined (P < 0.05), except for the pN3 categories (P = 0.178). However, no such significant differences were observed in the subgroup analyses of the LNR and LODDS staging systems (P > 0.05), except for the category of LNR0 (P < 0.001).

Furthermore, we also drew the ROC curves of these three LN staging systems in patients with different lymphadenectomies. In patients with 15 LNs or more examined, the corresponding AUCs for pN, LNR, and LODDS were 0.703 (95% CI 0.665–0.742), 0.716 (95% CI 0.678–0.754), and 0.733 (95% CI 0.696–0.770), respectively, and the difference was not significant (P = 0.312). In patients with less than 15 LNs examined, the corresponding AUCs for pN, LNR, and LODDS were 0.655 (95% CI 0.620–0.689), 0.660 (95% CI 0.626–0.695), and 0.680 (95% CI 0.649–0.714), respectively, and no significant difference in AUC was found in these three LN staging systems (P = 0.405).

## Discussion

An accurate cancer staging classification should ideally be widely accepted among surgeons, oncologists, and other physicians^15^. Moreover, it should also provide the clinician with information for the planning of treatment and evaluating the treatment results between different institutions and areas^16^.

From the 7th edition of the UICC/AJCC TNM staging system for esophageal carcinoma that had been used in 2010, the pN stage was classified according to the absolute number of involved LNs, rather than the simple classification of absent (pN0) or present (pN1) in the previous editions. Although the new pN stage was found to provide more accurate prediction of survival than the previous versions, this number-based pN stage still had some deficiencies^4,12,17–21^. The most important point was that stage migration usually occurred in this pN stage in patients with a small number of LNs examined^22^.

The number of LNs examined could vary significantly among different patient cohorts due to the different extents of lymphadenectomy. Thus, new prognostic nodal parameters were required to compensate for the deficiencies in these number-based pN stages. Previous studies have found that the LNR and LODDS stage might be superior to the pN stage because they were not significantly affected by the total number of LNs examined^3–11^, and some studies even found that LODDS might have better prediction of prognosis than the LNR^6–8^. However, controversy still exists^23^, and neither the LNR nor the LODDS stage has accurately and widely accepted criteria. Moreover, few studies have evaluated these two LN staging systems in ESCC^12,17–19^.

In the current study, we used a large patient cohort with ESCC to compare the prognostic value of three LN staging systems (pN, LNR and LODDS). In order to minimize the impact of the pT stage on survival, we enrolled patients with a single pT3 stage for analyses, which consisted of the largest proportion of patients with ESCC in our study (38.8%, 1667/4298). All three LN staging systems were found to be significantly correlated with survival in univariate and multivariate analyses, and the corresponding AUC also showed that none of them differed significantly in predicting survival, indicating that they could be used for prognostic assessment in ESCC.

However, when we analyzed the survival of patients in each pN and LNR classification stratified by the LODDS, significant differences in survival were always found, with the exception of pN3, LNR2, and LNR3. However, survival was highly homologous when the LODDS classification was stratified by the pN or LNR. Moreover, as the definition of the LNR0 category was the same as the pN0 category, both the pN and LNR staging systems could not discriminate the survival differences among patients with no LN metastasis. Due to its unique statistical characteristics, LODDS was the only LN staging system that could discriminate survival differences in patients without LN metastasis. All of these results suggested that LODDS might be superior to the other two LN staging systems.

The findings in our study that the LNR and LODDS staging systems could more accurately predict survival than the pN staging systems in patients with inadequate lymphadenectomy were consistent with previous studies^3–11^. In our study, we found that in most of the pN categories, better prognosis would always be found in patients with more extensive lymphadenectomy. However, survival was more homologous when subdividing the LNR and LODDS staging systems based on the extent of lymphadenectomy, except for the category of LNR0, which had the same definition as the pN0 category. These results did not mean that the LNR and LODDS staging systems were not influenced by the examined LN number. Theoretically, more extensive lymphadenectomies would always lead to the potential for better staging, not only for pN staging but also for LNR and LODDS staging. The corresponding AUCs for the LNR and LODDS staging systems in patients with adequate lymphadenectomy were higher than those in patients with inadequate lymphadenectomy, indicating that the accuracy of the LNR and LODDS staging systems was also positively correlated with the number of LNs examined. The superiority of prognosis assessment for the LNR and LODDS staging systems was that the influence of the number of LNs examined on them was smaller than that of the pN staging system^23^.

Our study has some limitations. Firstly, this was a retrospective study from a single center. The retrospective nature may undermine the power of our study. Secondly, the patients enrolled in our study were from a long period with different surgeons and pathologists. As no widely accepted criteria have been established for LNR and LODDS staging for ESCC, whether our results can be applied to other studies still needs to be confirmed. Thirdly, all of our patients were treated with primary surgery without neoadjuvant therapy. Few studies have concerned on the topic of the LNR and LODDS staging systems in patients with ESCC who received neoadjuvant therapy. Whether the staging systems presented in our study could be used for prognostic assessment in patients with pT3 ESCC who received neoadjuvant therapy is still controversial. We think that further multicenter, prospective studies are required to identify widely accepted criteria for LNR and LODDS staging in ESCC.

In conclusion, all three staging systems could be used for prognostic assessment in ESCC. However, the LNR and LODDS staging systems could more accurately predict survival than the pN staging system in patients with inadequate lymphadenectomy, and LODDS might be superior to the other two LN staging systems due to its unique statistical characteristics. Further studies are required to examine our findings and identify widely accepted criteria for LNR and LODDS staging in ESCC.
